# Treatment of Dupuytren Contracture Recurrence After Surgery With Collagenase Clostridium Histolyticum: A Retrospective Multicenter Series

**DOI:** 10.1016/j.jhsg.2025.100919

**Published:** 2026-01-20

**Authors:** Clayton A. Peimer, Marie A. Badalamente, Philip Blazar, Keith A. Denkler, William Dzwierzynski, Mark Elzik, F. Thomas D. Kaplan, Jason A. Nydick, Gary M. Pess, James Verheyden, Mark A. Vitale, Jeffrey Andrews, Qinfang Xiang, David Hurley, Lawrence C. Hurst

**Affiliations:** ∗ Department of Orthopedic Surgery, Warren Alpert Medical School, Brown University, Providence, RI; † Department of Orthopedics, Stony Brook University Medical Center, Stony Brook, NY; ‡ Department of Orthopedic Surgery, Brigham and Women’s Hospital, Boston, MA; § Division of Plastic and Reconstructive Surgery, University of California San Francisco, San Francisco, CA; ‖ Department of Plastic Surgery, Medical College of Wisconsin, Milwaukee, WI; ¶ South Orange County Orthopedics, Inc., Mission Viejo, CA; ∗∗ Indiana Hand to Shoulder Center, Indianapolis, IN; †† Florida Orthopedic Institute, Tampa, FL; ‡‡ Central Jersey Hand Surgery, Eatontown, NJ; §§ The Center, Bend, OR; ‖‖ ONS Foundation for Clinical Research and Education, Greenwich, CT; ¶¶ Endo USA, Inc., A Keenova Therapeutics Company, Malvern, PA

**Keywords:** Collagenase clostridium histolyticum, Dupuytren contracture, Medical records, Recurrence, Retrospective studies

## Abstract

**Purpose:**

Dupuytren contracture (DC) is a fibroproliferative disorder characterized by collagen deposition in the palmar fascia. Treatment options include collagenase clostridium histolyticum (CCH) injection and surgery; however, DC frequently recurs after primary therapy. We hypothesized that CCH treatment could be effective and well tolerated for the treatment of contracture recurrence for patients unwilling to undergo reoperation or at high risk for complications.

**Methods:**

This Phase 4, multicenter, noninterventional, retrospective study analyzed medical records from 10 clinical centers in the US. Patients were treated with CCH for DC recurrence ≥6 months after previously successful surgical correction performed between January 1, 2010, and August 15, 2020. Primary end points were the measured joint contracture change from baseline, at first and last clinical evaluation within 12 months of CCH treatment of metacarpophalangeal (MP) and proximal interphalangeal (PIP) joint contractures. Secondary end points were “clinical success” (percentage of joints with reduction in contracture to 0° to 5°) and adverse events.

**Results:**

Of 113 patients screened, 101 were analyzed (mean age, 64.1 years; 75% men). Median time to DC recurrence was 36.0 months. A total of 144 treated joints were analyzed (MP, *n* = 64; PIP, *n* = 75; unspecified, *n* = 5). Overall mean (SD) baseline contracture was 52° (21°) (MP, 43° [19°]; PIP, 61° [20°]). Mean (SD) improvement of contracture from baseline at last evaluation was 38° (21°) for all joints (MP, 36° [17°]; PIP, 41° [24°]), with 58% of joints having clinical success (MP, 75%; PIP, 43%). All skin tears (20 events in 19% of patients) resolved spontaneously, with 50% resolving in ≤21 days; there was one flexor profundus rupture that did not require secondary reconstruction.

**Conclusions:**

This retrospective analysis indicates that CCH treatment is an effective and well tolerated nonsurgical option for recurrent postsurgical contracture, with results comparable to patients without prior surgery.

**Type of study/level of evidence:**

Therapeutic III.

Dupuytren contracture (DC), or Dupuytren disease, is a proliferative disorder affecting 0.3% to 37% of US adults,^1^ primarily men.^2^ Characterized by thickening and shortening of the fascial bands in the palm and fingers, it leads to flexion contracture.^3^^,^^4^ Dupuytren contracture commonly involves the metacarpophalangeal (MP) and proximal interphalangeal (PIP) joints of the ring and little fingers but may affect any digit.^5^ Although the etiology remains unclear,^6^ potential risk factors include family history, diabetes, and heavy alcohol consumption.^1^, ^2^, ^3^^,^^7^, ^8^, ^9^ Dupuytren contracture can severely impair hand function, resulting in difficulties with everyday activities. Consequent decline in quality-of-life causes patients to seek treatment.^10^, ^11^, ^12^

Treatment options for DC include surgical excision (fasciectomy) or cord transection (fasciotomy); percutaneous needle fasciotomy (PNF); or injection with collagenase clostridium histolyticum (CCH).^4^ Surgery remains the most commonly used option, with a recent retrospective US Medicare administrative claims study reporting 62% of treated patients underwent fasciectomy compared to 29% who received CCH as primary treatment from 2011 to 2017.^4^^,^^13^

Regardless of treatment modality, DC recurrence (increase of flexion contracture by ≥20°),^14^ occurs after all treatments. Not all patients who experience a “recurrence” choose repeat treatment, especially if surgery is the only treatment option offered.^15^ Additionally, surgical treatment of recurrence can be more difficult because of scarring and postsurgical changes in anatomy, potentially resulting in increased local complications and risk of finger amputation with reoperation.^15^

Although efficacy of CCH in treating DC has been demonstrated in Phase 3, randomized, controlled trials and real-world clinical series, few studies have reported CCH use for recurrent contracture after initial surgery.^14^^,^^16^, ^17^, ^18^, ^19^ This Phase 4 study assessed the effectiveness and safety of CCH treatment for recurrent DC after surgery. We hypothesized that CCH treatment for recurrent DC could be effective and well tolerated in patients with previously successful surgical treatment.

## Materials and Methods

### Study design

This Phase 4, multicenter, noninterventional, retrospective review of medical records aimed to collect data from up to 20 sites in the US to collect >100 treated cases. To improve generalizability and avoid surgeon bias, each site initially could submit up to 10 patient charts. Additional records could be submitted if the site had not met the limit, and 100 records had not been collected after the initial 30-day collection period. Data from eligible cases were recorded and listed.

This study was conducted with the ethical principles originating from the Declaration of Helsinki, and in full compliance with the International Council for Harmonization E6 and Guidance on Good Clinical Practice guidelines. Confirmation of the exempt status for this study was obtained by Endo Pharmaceuticals, Inc., from a central institutional review board (IRB; Sterling IRB). Additional institutional review board approvals required by investigators’ affiliated institutions were obtained by the study physician at each site. No employee or consultant of the sponsor had access to any medical records or identifiable patient data.

### Patient population

Recurrent contracture was defined as a functionally considerable increase in flexion contracture of ≥20° that prompted a patient to seek care after previous successful surgery.^14^ Patients’ clinical data were included if they had a diagnosis of recurrent DC at ≥6 months after a previously successful surgical correction (ie, fasciectomy, fasciotomy, or PNF), and then received CCH treatment for recurrence between January 1, 2010, and August 15, 2020. Patients’ data were excluded if postsurgical/pre-CCH (“baseline”) joint measurement and/or post-CCH measurements were missing. For efficacy analyses, CCH-treated joints with baseline angle measurements <20° were excluded.

### Data collection

Data elements collected from patient records included: demographic characteristics, medication use, medical history, DC treatment history, and adverse events (AEs) with a start date on or after the first CCH treatment to final evaluation (Appendix S1, available online on the Journal’s website at https://www.jhsgo.org.). Adverse events of special interest included skin tear at or immediately after joint manipulation, flexor tendon or pulley tear, infection at the injection site, and neurovascular injury.

No data beyond what was available in patients’ medical records were collected; study physicians were responsible for ensuring medical records were complete, accurate, and comprehensive. Study physicians provided missing information by email for required but absent data elements.

### Study measures

The primary end points were the measured joint contracture change from baseline, at the first post-manipulation evaluation and at the last clinical evaluation (ie, last recorded angle of affected joint) within 12 months of CCH treatment. Secondary end points were “clinical success”, defined as the percentage of joints with a resulting contracture of 0° to 5° measured at first post-manipulation and last recorded evaluation, and safety, assessed by the percentage of patients with skin tears following initial manipulation and by the number of patients with AEs of special interest.^14^^,^^18^

### Statistical analysis

Descriptive summaries included frequency tables for categorical response variables and number, mean, SD, minimum, and maximum for continuous variables.

## Results

### Demographics and clinical characteristics

Records were collected from 10 sites between March 19, 2021, and August 15, 2021. Of 113 medical records screened, 101 patients were included in the analyses. Mean age was 64.1 years (range, 26–87); 75% were men, 80% were White, and 91% were right-handed. Demographics and medical history are found in the Table 1.

**Table 1:** Demographic and Medical History

Parameter	Patients (*N* = 101)
Mean age, y (SD)	64.1 (9.9)
Range	26–87
Sex, *n* (%)	
Male	76 (75)
Female	25 (25)
Race, *n* (%)	
White	81 (80)
Black	1 (1)
Not reported	19 (19)
Dominant hand, *n* (%)	
Right	92 (91)
Left	4 (4)
Not reported	5 (5)
Alcohol use, *n* (%)	
Never	25 (25)
Current	67 (66)
Former	4 (4)
Not reported	5 (5)
Tobacco use, *n* (%)	
Never	60 (59)
Current	5 (5)
Former	24 (24)
Not reported	12 (12)
Diabetes type I, *n* (%)	
Yes	5 (5)
No	96 (95)
Not reported	0 (0)
Diabetes type II, *n* (%)	
Yes	8 (8)
No	93 (92)
Not reported	0 (0)
Family history of DC, *n* (%)	
Yes	22 (21.8)
No	54 (53.5)
Not reported	25 (24.8)
Plantar fibromatosis (Ledderhose disease), *n* (%)	
Yes	13 (13)
No	77 (76)
Not reported	11 (11)
Peyronie’s disease,∗*n* (%)	
Yes	3 (4)
No	60 (79)
Not reported	13 (17)

∗ Summarized for men only. Percentages are based on the total number of men in the study.

Despite most patients being right-hand dominant, contractures equally affected both hands (left, 50.5%; right, 49.5%; Table 2). Three of 101 patients received treatment for both hands; all three patients received treatment for their left hand first.

**Table 2:** Previous Treatment History – First Treated Hand

Parameters	Patients (*N* = 101)
Treated hand, *n* (%)	
Right	50 (49.5)
Left	51 (50.5)
Number of surgeries before CCH treatment, *n* (%)	
1	77 (76.2)
2	19 (18.8)
≥3	4 (4.0)
Unknown∗	1 (1.0)
Previous treatment type, *n* (%)	
Needle aponeurotomy	23 (23)
Fasciotomy	7 (7)
Fasciectomy	70 (69)
Not reported	1 (1)
Time since most recent previous surgical treatment, months†	
*n*	85
Mean (SD)	61.9 (70.1)
Median	39.7
Range‡	3.1–515.9
Time to DC recurrence, months	
*n*	101
Mean (SD)	49.8 (66.1)
Median	36.0
Range	6–474

∗ Patient confirmed having surgical treatment but was unable to determine number of surgeries received.

† Time since most recent previous surgical treatment (months) was defined as date of initial postoperative CCH treatment minus the previous surgical treatment date.

‡ Two patients had CCH treatment approximately 3 months since their most recent surgical treatment date.

Most patients (77%) had undergone one surgical procedure before CCH treatment, with the remaining 23% reporting ≥2 prior surgeries (Table 2). Prior surgeries included fasciectomy (69%), needle aponeurotomy (23%), and open fasciotomy (7%); 1 patient had unspecified surgical type. Median (range) time from initial surgery to treatment of recurrence with CCH was 36.0 (range, 6–474) months.

### Efficacy of CCH treatment

**Figure 1:**
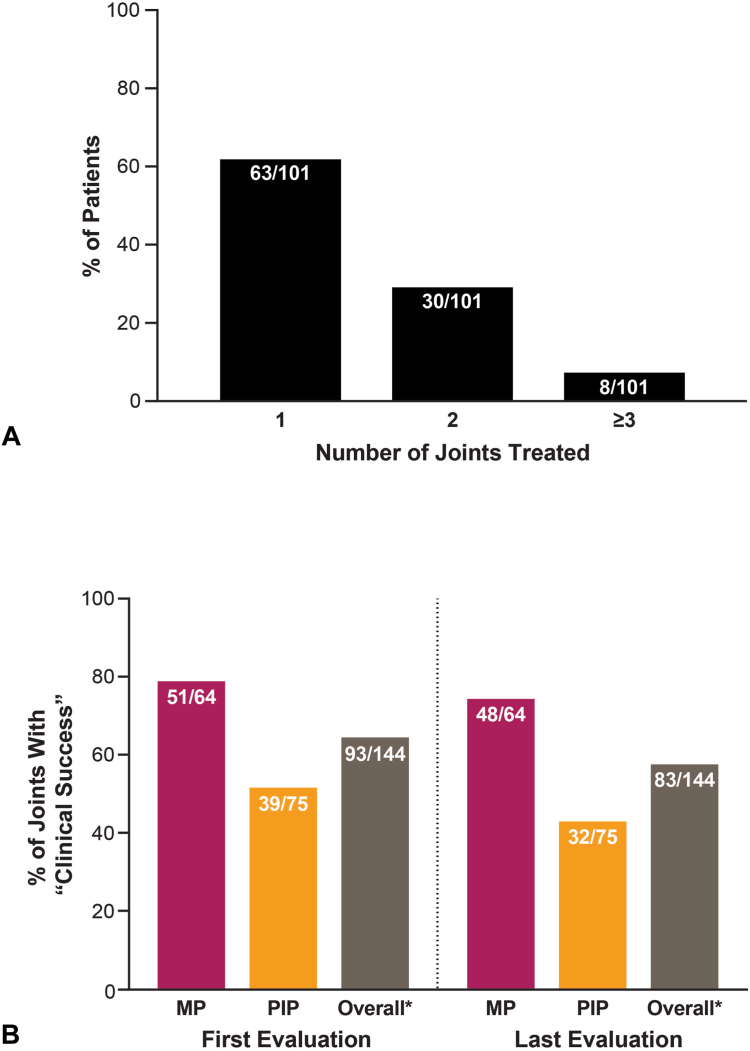
Proportion of patients based on: **A** number of joints treated and **B** “clinical success” (reduction of contracture to within 0°–5° after CCH treatment). Values above *bars* represent n/N: number of patients/total study population (**A**); number of joints showing “clinical success”/total number of joints treated (**B**). ∗Five patients, whose treated joints were not reported, were included in the total number of joints but were not included in analyses of specific (MP or PIP) joints. CCH, collagenase clostridium histolyticum; MP, metacarpophalangeal; PIP, proximal interphalangeal.

Sixty-two percent of patients had one joint treated with CCH, 30% had two joints treated, and 8% had ≥3 joints treated (Fig. 1). In total, 144 treated joints were analyzed (MP, *n* = 64; PIP, *n* = 75; Table 3). During chart review, five records did not record the treated joint (MP or PIP) and, thus, were unspecified. Unspecified joints were included in the overall number of joints but not in the analyses of specific joints.

**Table 3:** Observed Contracture and Contracture Change From Baseline (in °)

	Mean Degree (°) of Contracture (SD)∗		
	MP (*n* = 64)	PIP (*n* = 75)	Overall† (N = 144)
Baseline‡	43 (19)	61 (20)	52 (21)
Minimum, maximum	20, 80	25, 100	20, 100
First evaluation§	6 (14)	13 (17)	10 (16)
Minimum, maximum	0, 75	0, 60	0, 75
Change from baseline to first evaluation	−37 (19)	−48 (22)	−43 (21)
Minimum, maximum	−80, 30	−90, −5	−90, 30
Last evaluation‖	7 (13)	20 (22)	14 (19)
Minimum, maximum	0, 60	0, 85	0, 85
Change from baseline to last evaluation	−36 (17)	−41 (24)	−38 (21)
Minimum, maximum	−80, 0	−90, 15	−90, 15

∗ Patients with >3 joints treated were excluded from analysis.

† Five patients, whose specific treated joints were not reported, were included in the total number of joints but not in analyses of specific (MP or PIP) joints.

‡ Baseline was defined as the last postsurgical joint measurement before CCH treatment.

§ The first recorded joint measurement post-CCH treatment. If multiple CCH treatments were given, then the first recorded measurement after the last CCH treatment.

‖ The last recorded joint measurement within 12 months of the postsurgical dose of CCH treatment.

**Figure 2:**
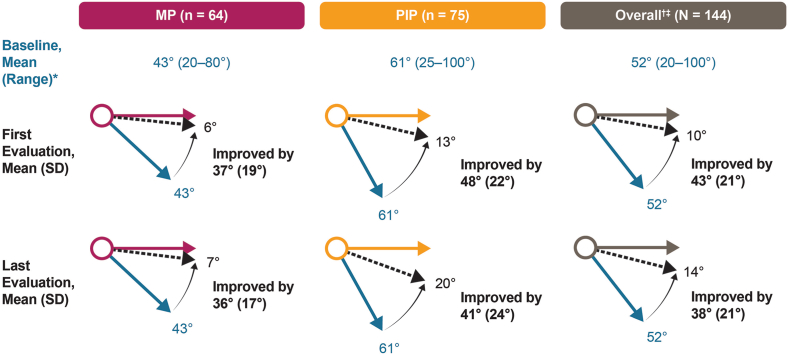
Observed contracture and contracture change from baseline. *Blue arrows* represent mean baseline contracture, and *dashed arrows* represent mean contracture at first and last evaluations in degrees. ∗Baseline was defined as the last postsurgical joint measurement before CCH treatment. **^†^**Patients with >3 joints treated were excluded. **^‡^**Five patients whose treated joints were not reported were included in the total number of joints but were not included in analyses of specific (MP or PIP) joints. CCH, collagenase clostridium histolyticum; MP, metacarpophalangeal; PIP, proximal interphalangeal.

Mean (SD) baseline contracture was 52° (21°), with PIP joints showing a higher degree of recurrent contracture (61° [20°]) compared to MP joints (43° [19°]). At first evaluation after CCH treatment, the mean range of motion (SD) increased by 43° (21°) from baseline for all joints (Fig. 2). Treated PIP joints demonstrated an increase in extension of 48° (22°), while MP joints showed an increase of 37° (19°), from baseline. At their final evaluation (last recorded joint measurement ≤12 months after CCH treatment), the overall mean (SD) improvement of contracture was 38° (21°). Improvement from baseline for PIP joints was 41° (24°), whereas MP joints remained essentially unchanged from first post-manipulation evaluation (36° [17°]).

### Clinical success

Clinical success, defined as a reduction of joint contracture to within 0° to 5°, for all joints was 65% and 58% after the first and last evaluations, respectively (Fig. 1B). Treated MP joints had higher clinical success rates than PIP joints, with 80% and 75% of MP joints demonstrating success at first and last evaluations, respectively, compared to 52% and 43% of PIP joints.

### Safety

Thirty-six (36%) patients were reported to have 38 AEs considered related to CCH treatment (see Table S1 for complete list of AEs, available online on the Journal’s website at https://www.jhsgo.org). Of special interest, 20 skin tears occurred after manipulation in 19 patients. Half of the skin tears needed ≥21 days to fully heal; none required secondary surgery or skin graft, and there was no evidence that the MP or PIP joint was more/less prone to skin tears after CCH treatment (Table S2, available online on the Journal’s website at https://www.jhsgo.org.). There was one rupture of the flexor digitorum profundus tendon considered likely because of CCH treatment, but this AE was not treated surgically; additional information as to which finger was not reported.

## Discussion

This study evaluated outcomes in patients with recurrent DC who were initially treated with fasciectomy or PNF and who then received retreatment with CCH. Recurrence after primary treatment remains an unsolved problem regardless of the initial treatment performed, yet despite the likelihood of recurrence, studies comparing the outcomes of different retreatment procedures are rare.^20^ To the best of our knowledge, this is first large study of CCH retreatment for functionally significant recurrent contractures ≥20° after previously successful surgical correction.

At their last evaluation, patients included in this study had an overall mean contracture improvement of 38°, with clinical success observed for 58% (all treated joints). Two patients had CCH treatment approximately 3 months after their most recent surgical treatment date. This is an uncommonly short time to recurrence. Both patients improved after treatment with one patient achieving clinical success at the first and last post-treatment evaluations.

Our results agree with previous reports of CCH for recurrent DC. A study of patients treated with CCH for primary DC who then received additional collagenase injections for recurrent contractures found that 3 months after retreatment, 65% of the MP and 45% of PIP joints had achieved clinical success.^21^ A case report of CCH for recurrent DC following two previous fasciectomies, including a skin graft, and PNF, reported that 3 months after CCH injection the patient’s PIP contracture was reduced from 90° to 40° and distal interphalangeal joint contracture was reduced from 30° to 0°.^22^ Our results also are consistent with a pooled analysis of 1,082 patents enrolled in 12 randomized or open-label CCH clinical trials which found that the efficacy of CCH was unaffected by prior surgical treatment.^23^ At 30 days after CCH injection, approximately equal proportions of joints in hands with prior surgery versus those without demonstrated reduction of recurrent contracture to ≤5°.

Adverse events were consistent with prior reports of CCH in patients without prior surgical treatment. The most commonly reported AE in this study was skin tears (incidence 19%), comparable to the reported incidence of 22% to 26%.^24^^,^^25^ Tendon rupture (1 event in 1 patient) also was recorded in this study; past studies have reported tendon ruptures in 1–3 patients.^16^^,^^19^^,^^26^

Outcomes of patients with recurrent contractures treated by collagenase injection can be compared to those patients treated surgically, although it should be noted that the variety of surgical techniques were used and variable definitions of recurrence make direct comparisons difficult. A prospective study of 43 patients who underwent fasciectomy as a primary treatment and also as retreatment for recurrent DC reported that mean MP and PIP contractures were reduced by 22° and 43°, respectively, 61–77 months after the repeat procedure, similar to the results reported here.^27^ A chart review of 54 surgical retreatments for MP and PIP contractures in 33 patients, including partial fasciectomies, dermofasciectomies, and radical fasciectomies reported that after a mean of 30 days postoperatively, MP contractures improved from 10° to 41° and PIP contractures from 38° to 49°.^28^ Finally, a retrospective study in 159 patients treated with PNF for primary and recurrent DC described MP and PIP contracture improvements of 20° and 15°, respectively, improvements approximately 50% of those reported here.^29^

Risks of complications, such as vascular injury, nerve injury, necrosis, and infection, are greater with repeat fasciectomy than primary surgery.^30^^,^^31^ For example, rates of digital artery injury are greater with recurrent surgery (11% to 30%) than primary surgery (1% to 3%). In addition, AEs of nerve laceration are observed in 2% to 5% of retreated patients,^31^ with one study reporting tactile deficits in 68% of reoperated fingers and anesthetization in 11%.^32^ A large database analysis of 121,488 patients who underwent surgery for DC found that the incidence of amputation in primary surgery of 0.3% rose to 1.5% for the first reoperation for recurrence, and was 8% in patients who had initially undergone dermatofasciectomy followed by limited fasciectomy.^15^

There are several limitations to consider in our findings. As a retrospective chart review, the data are limited in terms of collection and availability; collection of some raw data as open field text, such as time to recurrence, may lead to data discrepancies. Follow-up was short and heterogeneous, with the defined primary end points considering clinical evaluations of ≤12 months after treatment, since we sought to determine if CCH was useful for recurrent DC postoperatively. Data collection across multiple centers introduced variable recording; furthermore, there is no standardized protocol for measuring contracture, or consensus on skin tear recording, the most common complication seen beyond transient swelling, ecchymosis, and pain. Lastly, patient selection criteria for treatment were determined by individual surgeons and not predetermined and standardized, so applicability to patients with severe scarring, prior skin grafts, and other complex situations cannot be entirely addressed.

Strengths of this study include the collection of real-world evidence, which reflects current clinical practice and outcomes. Additionally, data collection across 10 US centers and from multiple surgeons allows for generalizability to a broad population of patients with recurrent DC.

Based on the results of this study, we believe CCH treatment for recurrent contracture postoperatively is effective and well tolerated. Choice of follow-up procedure should be determined in part by the initial procedure and its success and the severity of the recurrent contracture, and patient preference.^20^^,^^31^ Depending on contracture severity, surgeons and patients may hesitate to reoperate for recurrent DC because of the higher risk of postoperative complications, such as neurovascular injuries. Although difficult to quantify, postoperative scarring from prior surgery/surgeries may lead to increased complexity and poor functional outcomes after reoperation, including instances of diminished touch and protective sensibility, and complete anesthesia.^32^^,^^33^ While factors, such as prior skin grafts or severe scarring, may preclude some cases from treatment, CCH generally is an effective nonsurgical option for these patients with recurrent disease after surgery. Patient preference is an important consideration for the follow-up approach as well, as some patients with recurrence may not wish to undergo another operation and a lengthy postoperative interval of disability.

Taken together, our findings indicate that CCH treatment is an effective and well tolerated option for recurrent postoperative DC, with results comparable to patients without prior surgical treatment as well as to those who underwent surgery for recurrent contractures.

## Conflicts of Interest

Dr Peimer reports personal fees from Endo Pharmaceuticals, Inc., outside the submitted work. In addition, Dr Peimer has a patent broadly relevant to the work that was issued before study onset. Dr Blazar reports personal fees from Endo Pharmaceuticals, Inc., outside the submitted work. Dr Denkler has served on an advisory board for Endo Pharmaceuticals, Inc., in 2022 and performed a clinical trial chart review for Endo Pharmaceuticals, Inc., in 2021. Dr Dzwierzynski reports grants from Endo Pharmaceuticals, Inc., during the conduct of the study. Dr Elzik reports personal fees from Endo Pharmaceuticals, Inc., during the conduct of the study; personal fees from Endo Pharmaceuticals Inc., outside the submitted work. Dr Kaplan reports grants from Endo Pharmaceuticals, Inc., outside the submitted work. Dr Pess reports receiving personal fees for serving on the speakers’ bureau for Endo Pharmaceuticals Inc., outside of the submitted work. Dr Verheyden reports personal fees from Endo Pharmaceuticals, Inc., during the conduct of the study; personal fees from Endo Pharmaceuticals Inc., outside the submitted work. Dr Vitale has served on an advisory board for Endo Pharmaceuticals, Inc., in 2022. Mr Andrews, Drs Xiang, and Hurley are employees of Endo Pharmaceuticals, Inc., the sponsor of the study. No benefits in any form have been received or will be received by the other authors related directly to this article.
