# Supplementary material for: Treatment of Dupuytren Contracture Recurrence After Surgery With Collagenase Clostridium Histolyticum: A Retrospective Multicenter Series
Source: J Hand Surg Glob Online. 2026 Jan 20;8(2):100919. doi: 10.1016/j.jhsg.2025.100919 (PMC12859253; doi:10.1016/j.jhsg.2025.100919)
Supplement: Supplementary Material [file mmc1.docx]

**Appendix S1:**The following data elements were collected from patient records:

- Demographic characteristics
  - Birth year
  - Sex
  - Race/ethnicity
  - Dominant hand
  - Tobacco use
  - Alcohol use
- Medication use
- Medical history
  - Family history of DC
  - Diabetes
  - Dupuytren diathesis (personal history of plantar fibromatosis, finger knuckle pads, or Peyronie’s disease)
- Dupuytren disease treatment history
  - Affected hand/finger(s)/joint(s) (eg, MP or PIP)
  - Previous treatment types
  - Previous surgical treatment date
  - Months to DC recurrence
  - Angle of affected joint prior to CCH manipulation
  - CCH treatment
    - Date of initial postsurgical dose
    - Single or double dose
  - Angle of joint post-CCH manipulation
  - Secondary/ancillary treatments (splint use, skin graft)
  - Angle of affected joint at last clinical evaluation ≤12 months post-CCH manipulation
- Adverse events (AEs) from time of first CCH treatment date out to 12 months
  - AEs of special interest
    - Skin tear at, or immediately after, manipulation
    - Tendon/flexor pulley tear
    - Infection at the site of injection
    - Neurovascular injury

**Table S1:** Overall Summary of AEs

| Parameter | Overall  (N = 101) | |
| --- | --- | --- |
|  | n (%) | Events |
| Number of patients reporting CCH-related AEs*: | | |
| At least 1 CCH-related AE | 36 (36) | 38 |
| At least 1 CCH-related AE leading to interruption/discontinuation of CCH | 0 (0) | 0 |
| At least 1 CCH-related AE of special interest^†^ | 19 (19) | 20 |
| SOC^‡^ PT^‡^ |  |  |
| Blood and lymphatic systems Lymphadenopathy | 1 (1) 1 (1) | 1 1 |
| General disorders and administration site conditions Swelling | 2 (2) 2 (2) | 2 2 |
| Injury, poisoning, and procedural complications Skin tear Tendon rupture Contusion | 21 (21)  19 (19)  1 (1) 1 (1) | 22 20 1 1 |
| Musculoskeletal and connective tissue disorders Limb discomfort | 1 (1) 1 (1) | 1 1 |
| Skin and subcutaneous tissue disorders Ecchymosis | 12 (12) 12 (12) | 12 12 |

Note: AEs are those with a start date on or after the initial postsurgical CCH treatment through 1 year after the initial CCH treatment.

*CCH-related AEs are those reported as having possibly or probably related to CCH. Missing relationships were considered as related. Total number of AEs reported includes the same AE occurring multiple times for a patient being counted at each occurrence. Percentages were based on the overall total number of AEs.

^†^AEs of special interest include skin tear – at or immediately after – manipulation, tendon/flexor pulley tear, infection at the site of injection, and neurovascular injury.

^‡^SOC and PT were coded using the MedDRA dictionary (version 24.0). If multiple AEs were reported within a given SOC and/or PT, only 1 event was counted per patient.
AE, adverse event; CCH, collagenase clostridium histolyticum; SOC, System Organ Class; PT, Preferred Term.

**Table S2**: Patients With Skin Tear During CCH Treatment

| For any CCH-Treated Joint,* Did the Patient Experience a Skin Tear?,  n (%) | MP  (n = 64) | PIP  (n = 75) | Overall^†^  (N = 144) |
| --- | --- | --- | --- |
| Yes | 17 (27) | 17 (23) | 35 (24) |
| No | 47 (73) | 58 (77) | 109 (76) |

*Patients with >3 joints treated were excluded from analyses.

^†^Five patients, whose treated joints were not reported, were included in the total number of joints but were not included in analyses of specific (MP or PIP) joints.
CCH, collagenase clostridium histolyticum; MP, metacarpophalangeal; PIP, proximal interphalangeal.
